# Anti-recoverin Antibody-Associated Post-acute COVID Vaccination Syndrome After BNT162b2 in HLA-B27-Positive Spondylarthritis: A Case Report

**DOI:** 10.7759/cureus.66881

**Published:** 2024-08-14

**Authors:** Josef Finsterer

**Affiliations:** 1 Neurology, Neurology and Neurophysiology Center, Vienna, AUT

**Keywords:** adverse reaction, vertigo, headache, sars-cov-2 vaccination, recoverin antibodies

## Abstract

Retinopathy, small fiber neuropathy (SFN), and encephalopathy associated with recoverin antibodies have not been previously reported as side effects of BNT162b2 vaccination in a patient with HLA-B27-associated spondylarthritis.

The patient is a 47-year-old male with a 10-year history of HLA-B27-associated spondylarthritis without recurrence, who developed acute and post-acute COVID vaccination syndrome (ACVS/PACVS) after the first dose of the BNT162b2 vaccine. The PACVS manifested as cerebral disease, eye disease, and SFN. Two years after the onset of the adverse effects, recurrent elevated recoverin antibodies were detected. Despite the administration of various treatments, most symptoms persisted for more than three years, and only a few interventions such as glucocorticoids, hyperbaric oxygen therapy, botulinum toxin, inuspheresis, and HELP (heparin-induced extracorporeal LDL precipitation) apheresis showed a transient beneficial effect.

In conclusion, this case offers an example of a collection of symptoms following SARS-CoV-2 vaccination (SC2V) in a patient with a specific autoimmune disorder and positivity for anti-recoverin antibodies. These clinical manifestations may be triggered by an exaggerated immune response known as multisystemic inflammatory syndrome in adults to SC2V. Clinicians should report other similar cases to determine if a pattern exists.

## Introduction

SARS-CoV-2 vaccinations (SC2Vs) are not free from adverse effects [1]. Side effects can manifest as acute COVID vaccination syndrome (ACVS) within hours or a few days after vaccination or as post-acute COVID vaccination syndrome (PACVS) several days or weeks after vaccination [2]. ACVS/PACVS can be mild to fatal and can occur at any age [1]. There is evidence that certain pre-existing conditions increase the risk of adverse reactions [3], one of which is immunological disorders [4]. Among these, rheumatologic diseases have occasionally been reported in association with PACVS [5].

Recoverin is a neuron-specific calcium-binding protein that is mainly found in the retina and the pineal gland [6]. Elevated antibodies against recoverin are usually associated with malignancy and manifest as a paraneoplastic syndrome, predominantly with retinopathy or uveitis [7], but occasionally also with cerebellar syndrome [8]. There is also evidence that anti-recoverin antibodies may be associated with autoimmune encephalitis (AIE) [9]. Retinopathy associated with anti-recoverin antibodies manifests as rapid visual impairment, night blindness, color loss, vitreous cells, and a flat or severely reduced electroretinogram (ERG) [7].

Small fiber neuropathy (SFN) affects a-delta and C-fibers and is clinically characterized by sensory disturbances, pain of variable distribution, including complex regional pain syndromes, and autonomic dysfunction [10]. SFN has been repeatedly reported as a side effect of SC2V with the BNT162b2 vaccine or the Moderna vaccine [11]. SFN as a side effect of SC2V in a patient with HLA-B27-positive spondylarthritis and positivity for anti-recoverin antibodies has, to the best of our knowledge, not been reported.

## Case presentation

The patient is a 47-year-old man, 185 cm tall, weighing 85 kg, who suffered from a scratchy throat and dizziness a few minutes after the first administration of BNT162b2 in April 2021, followed by shortness of breath, palpitations, drowsiness (brain fog), depersonalization, holocranic headache, and arterial hypertension (Table 1). These symptoms disappeared completely after about three days. His past medical history was positive for HLA-B27- and HLA-DR4-associated seronegative (rheumatoid factor-negative) spondylarthritis, which was first diagnosed in 2001 and treated with hydroxychloroquine (discontinued due to visual disturbances), methotrexate (MTX) for a few months (was ineffective), and sulfasalazine (2004 to 2011). He has not needed any treatment since 2011.

**Table 1:** Timeline of symptoms, most important results of instrumental examinations, and therapeutic consequences since the BNT162b2 vaccination BTX: botulinum toxin; HELP: heparin-induced extracorporeal LDL precipitation; CD: carotid ultrasound; ECG: electrocardiography; ERG: electroretinography; GBT: gabapentin; IF: interferon; MRA: magnetic resonance angiography; PCA: posterior cerebral artery; MRI: magnetic resonance imaging; MRV: magnetic resonance venography; PACVS: post-acute COVID vaccination syndrome; MTX: methotrexate; RV-ab: recoverin antibodies; SSEP: somatosensory evoked potential; NCSs: nerve conduction studies; repstim: repetitive nerve stimulation; SSR: sympathetic skin response; TCD: transcranial duplex sonography; US: ultrasound; VEP: visually evoked potentials; ^: normal.

Date	Symptoms/events	Examinations/results	Consequence/treatment
16.4.2021	SC2V, brain fog, dizziness, depersonalization, headache, palpitations, hypertension	No examinations commissioned	None, spontaneous recovery after 3 days
2.5.2021	Sudden headache, hypoesthesia left face, blindness, double vision, dizziness, chest pressure, palpitations	Neurological exam, cerebral MRI	None
Since then	Visual acuity decreased, floater images, disturbed color vision	Ophthalmological exam (refractive anomaly, normal funduscopy, normal OCT)	New spectacles
21.5.2021	Palpitations, daze, precordial pain, myocloni, hypertension, gait disturbance	Echocardiography and ECG	Bisoprolol, thomapyrin
6.7-9.7.2021, 20.7-22.7.2022	Persisting symptoms	Distal pallhypesthesia, MRI, MRV	Duloxetine, pregabalin, methylprednisolone in September 2021 beneficial
13.1.2022	Persisting symptoms	Cerebral MRI: non-specific spot right frontal, MRA: PCA aneurysm, cervical MRI: disc protrusions	HELP apheresis beneficial, hyperbaric oxygenation in February 2022 beneficial
9.5.2022	Persisting symptoms	Cardiologic exam, ECG, echocardiography, spike protein serum ­	Prednisolone 5 mg/d, diclofenac, desloratadine, cetirizine, rosuvastatin, vitamin D, candesartan
20.7-22.7.2022	Optic neuritis?	CSF protein­, VEPs, median SSEPs, tibialis SSEPs, carotid US, NCSs (tibial, peroneal, sural nerves), spike protein CSF and serum­, nucleocapsid protein negative	Methylprednisolone for 3 days
11.10.2022	Relapse of spondylarthritis?	FDG-PET: hypermetabolism occipital cortex bilaterally	None
28.10.2022	PACVS	Neurological exam	Venlafaxine
24.11.2022	Relapse of spondylarthritis?	Rheumatologist, lung X-ray, X-ray cervical spine (mild degeneration)	MTX (10 mg/w) prednisolone 10 mg/d, ASS, pantoprazole
12.3.2023	Persisting symptoms	Capillary microscopy and laser perfusion (microcirculatory deficit, reduced capillary blood flow), CD3 cells, TH1 response, TH2 shift, IFg, calprotectin	Recommended: naltrexone, aripiprazole, coenzyme Q10, L-glutathione, cromoglicic acid, montelukast, ketotifen, ozone therapy
20.3.2023	Persisting symptoms	Skin biopsy (reduced intra-epidermal nerve fiber density in the neck)	None
12.5.2023, 15.5.2023	Persisting symptoms	Ophthalmologic exam, corneal confocal microscopy (corneal fiber density)	Dexamethasone eye drops, cyclosporine eye drops
10.7.2023	Persisting symptoms	Cerebral MRI unchanged, MRA. Known aneurysm, cervical MRI: disc protrusions	None
2.8.2023	Suspected vasculitis	MRI, MRA, black blood, RV-ab +, pallhypesthesia, CD, TCD, repstim, optic nerve sheath US, SSR, SPA left suralis, SSEP, VEP, EMG, sudoscan	MTX, folic acid, GBT, prednisolone 5 mg/d
10.8.2023	SFN	Luria test, abnormal quantitative sensory testing, abnormal quantitative sudomotor axon reflex, B1, B6, B12, folic acid ­	Symptomatic therapy withdrawal of vitamin supplementation
25.8.2023	Persisting symptoms	MRI brain, cervical spine, plexus (unchanged to the previous exam)	None
18.9.2023, 19.9.2023	Persisting symptoms	ERG, funduscopy, Schirmer test (sicca syndrome)	Hylogel, evotears, ikervis
30.10.2023	Visual impairment	Ishihara test, stereotest abnormal, keratometry, eye pressure, pachymetry, Schober test, Amslertafel test, Scheimpflug image, corneal topography, OCT, funduscopy (TV, KZ, KS of vessels), OCTA (microcirculatory dysfunction)	Ozone therapy
15.11.2023	PCA aneurysm	Neurosurgical assessment	Wait and control
30.1.2024	Persisting symptoms	Cerebral MRI, unchanged to the previous exam, stenosis C5/6 left	None
8.2.2024	Persisting pain, visual impairment	RV-ab syndrome	Immunoadsorption recommended
July 2024	Persisting pain, visual impairment	None	Immunoadsorption, BTX

In May 2021, 16 days after the vaccination, the patient experienced sudden onset of headache radiating to the eyes, pain in the face, temples, back of the head, neck and a burning sensation in the mouth that lasted about four hours, hypesthesia in the left face and blindness for a few seconds, followed by double vision, focusing disorder, drowsiness, attention-deficit disorder, dizziness, chest pressure, and palpitations. One week later, he noticed increased sensitivity to light. Neurological examination, including a simple cerebral magnetic resonance imaging (MRI) scan, was unrevealing. Since this event, he noticed undulating but persistent headaches, brain fog, concentration problems, attention deficits, perceptual disturbances, pain in the neck temples, eyes, and eyelids, eye movement pain, persistent blurred vision, decreased visual acuity, blurring, floater images with bright backgrounds, and decreased twilight vision. Ophthalmologic examination showed normal extraocular movements and normal funduscopy. He needed new glasses, but they were of limited use.

In May 2021, five weeks after vaccination, he consecutively developed generalized myoclonus, especially before falling asleep, fine motor disturbances, lower limb weakness (MRC 5-), and gait disturbances. This was followed by palpitations, precordial pain, and high blood pressure. Electrocardiography (ECG), telemetry, carotid ultrasound, and echocardiography were inconclusive. Since this event, he noticed recurrent myoclonus, especially before sleep.

At a second neurological examination in July 2021, cerebral MRI and MR venography (MRV) were normal, but magnetic resonance angiography (MRA) incidentally revealed a right posterior cerebral artery (PCA) aneurysm of 1.6 mm. CSF examination revealed elevated protein levels and a disruption of the blood-brain barrier (BBB). Electroencephalography (EEG) was normal. The somatosensory evoked potentials (SSEPs) of the median nerve showed normal N20 latencies, and normal amplitudes, but an amplitude difference of >50% between the left and right sides. His symptoms were interpreted as psychosomatic, and duloxetine (10/2021-1/2022) and pregabalin (maximum 150 mg/day) were administered, but the symptoms did not improve. A treatment trial in September 2021 with methylprednisolone intravenously over five days led to a reduction in pain and an improvement in motor function, but an increase in brain fog. In October 2021, the patient suffered a mild SARS-CoV-2 infection, which resolved completely without treatment.

In April 2022, 12 months after the vaccination, he also developed a color vision disorder, more in the left eye than the right, so that blue, green, and red were seen as if through a filter. Unfortunately, no ophthalmologic examination was initiated at this time.

An examination for cardiac symptoms in May 2022 revealed arterial hypertension and atherosclerosis, which is why he was given candesartan and rosuvastatin.

A neurological examination in July 2022 for suspected optic neuritis (pain on eye movement) revealed a normal clinical examination, normal visual evoked potentials (VEPs), normal median and tibial SSEPs, and normal motor nerve conduction studies, including F-waves. CSF examination revealed a protein level of 60 mg/dl (n, 15-45 mg/dl), which was due to BBB dysfunction but was otherwise normal. Anti-SARS-CoV-2 spike antibodies were elevated to 11 E/ml in CSF and 40 E/ml in serum. Anti-SARS-CoV-2 capsid antibodies were negative. Interleukin-4 (Th2) and interleukin-1 were elevated and there was a Th1/Th2 shift. Methylprednisolone was administered intravenously for three days, which resulted in mild relief of eye pain but an increase in brain fog. In October 2022, a positron emission tomography (PET) computed tomography (CT) scan revealed non-specific meningeal calcification and hypermetabolism of the visual cortex in questionable arthritis or vasculitis. The rheumatologists suspected the persistence of the spike protein, which is why MTX (15 mg/day) and prednisolone were administered. In the same month, the patient suffered a second mild SARS-CoV-2 infection.

In February 2023, he received ozone therapy, and low-dose naltrexone, nattokinase, serapetase, fexofenadine, quercetin, clonidine, delimun, valacyclovir, alpha-lipoic acid, and N-acetylcysteine were administered without significant effect.

In March 2023, capillary microscopy of the nails revealed microbleeds, dilatation, and increased capillary branching, which is why a microcirculatory deficit and endothelialitis were diagnosed. Laser perfusion measurement revealed marked endothelial dysfunction, which is why acetylsalicylic acid, clopidogrel, and dabigatran were administered and four cycles of heparin-induced extracorporeal LDL precipitation were performed (Table 1). In addition, he received hyperbaric oxygen therapy, which reduced his symptoms by 90%. Skin biopsies in the same month revealed a presumably reduced intraepidermal nerve fiber density (IENFD) in the neck, but a normal IENFD density of 12.54 fibers/mm (n, >9.55 fibers/mm) in the thigh (Table 1).

**Figure 1:**
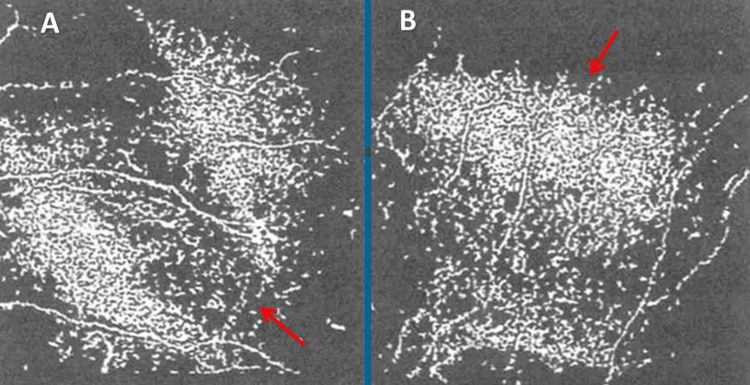
Corneal confocal microscopy in May 2023 shows reduced corneal nerve fiber density, reduced corneal nerve branch density, and reduced corneal nerve fiber length in the right (panel A) and left (panel B) eyes

A further examination of ocular symptoms in May 2023 revealed blepharitis, congested meibomian glands, telangiectasia of the cornea, and subepithelial fibrosis of the conjunctiva. Corneal confocal microscopy (CCM) revealed a corneal nerve fiber density of 12 mm^2^ (n, >24 mm^2^), a reduced corneal nerve branch density of 14/mm^2^ (n, >25/mm^2^), and a reduced corneal nerve fiber length of 9/mm^2^ (n, >15/mm^2^) (Figure 1). Dexamethasone and cyclosporine eye drops were administered.

The clinical neurological examination in May 2023 revealed reduced Achilles tendon reflexes, a non-specific tendency to fall in the Romberg and Unterberger test, and distal pallhypesthesia in the lower limbs. The patient reported a slowing of thought processes, a deterioration in dual-tasking, and being overwhelmed by new information.

During the investigation of suspected cerebral vasculitis in August 2023, a stable aneurysm of the right PCA was found on MRA, but the black blood sequences were normal. CSF examinations revealed elevated CSF proteins (563 mg/l (n, 200-400 mg/l)) and elevated antibodies against the spike protein in the CSF (Table 1). Anti-recoverin antibodies were determined for the first time and were positive in serum with a titer of 1:32 and in CSF (Table 2). VEPs, median and tibial SSEPs, repetitive nerve stimulation, and optic disc diameter were normal.

**Table 2:** Results of relevant blood/CSF tests carried out during three years between May 2021 and June 2024 GFAP: glial fibrillary acidic protein; NfH: neurofilament heavy chain; NfL: neurofilament light chain. *Determined four times. #Determined three times. $Determined two times.

Test	Date	Result
Hu	7.2.2024	Negative*
Ri	7.2.2024	Negative*
Yo	7.2.2024	Negative*
Ma/Ta	16.1.2024	Negative#
GAD65	7.2.2024	Negative$
Amphiphysin	7.2.2024	Negative#
CV2/CRMP-5	7.2.2024	Negative$
SOX1	7.2.2024	Negative$
Tr/DNER	7.2.2024	Negative#
Zic4	7.2.2024	Negative$
Titin/MGT30	7.2.2024	Negative$
PKCg	7.2.2024	Negative
ANAB	7.2.2024	<1:100
ANNA-3	8.8.2023	Negative$
AQP-4	8.8.2023	Negative
MOG	8.8.2023	Negative
NMDA	7.2.2024	Negative$
AMPA	7.2.2024	Negative$
GABA-b	7.2.2024	Negative$
LGI1	7.2.2024	Negative$
CASPR2	7.2.2024	Negative$
IgLON5	8.8.2023	Negative
ZIC4	8.8.2023	Negative
DPPX	7.2.2024	Negative
Anti-myelin	8.8.2023	Negative
CARPVIII	8.8.2023	Negative
mGluR1	8.8.2023	Negative
nGluR5	8.8.2023	Negative
GABA-a	8.8.2023	Negative
Rho GTPase activating protein 26	8.8.2023	Negative
GluRD2	8.8.2023	Negative
Flotillin1/2	8.8.2023	Negative
ITPR	8.8.2023	Negative
Homer3	8.8.2023	Negative
Neurochondrin	8.8.2023	Negative
Neurexin-3-alpha	8.8.2023	Negative
ERC1	8.8.2023	Negative
Sez6i2	8.8.2023	Negative
AP3B2	8.8.2023	Negative
Contactin I	8.8.2023	Negative
Neurofascin-155	8.8.2023	Negative
Neurofascin-185	8.8.2023	Negative
AT1A3	8.8.2023	Negative
KCNA2	8.8.2023	Negative
DA2R	8.8.2023	Negative
CKCL13 (CSF)	3.5.2024	Normal
CD3 cells	12.3.2023	Reduced
IL1	5.8.2022	Increased
IL4	5.8.2022	Increased
Th1 (IFg) response	28.12.2022	Increased
	15.3.2023	Reduced
Th1/Th2 (IL4)	28.12.2022	Reduced
	15.3.2023	Reduced
Cardiolipin antibodies	2.8.2023	Normal
Beta2-glycoprotein	2.8.2023	Normal
Neurotropic viruses	8.8.2023	Negative
Anti-SARS-CoV-2 spike (serum)	2.6.2022,	40IE
	3.5.2024	25 E/ml
Anti-SARS-CoV-2 spike (serum)	28.11.2022	>2500 U/ml (n, negative)
	2.8.2023	1005 U/ml (n, negative)
	9.10.2023	865 U/ml (n, negative)
Anti-SARS-CoV-2 IgG spike (serum)	2.8.2022	40 E/ml
	8.8.2023	27 E/ml
	17.1.2024	17 mg/ml
Anti-SARS-CoV-2 spike (CSF)	2.9.2022	11 E/ml
	8.8.2023	5 E/ml (n, negative)
	3.5.2024	5 E/ml (n, negative)
Anti-SARS-CoV-2 capsid IgG (serum)	2.6.2022	Negative
	28.11.2022	Positive
	4.3.2024	Negative
Anti-SARS-CoV-2 capsid IgG, IgM (serum)	28.12.2022	Negative
	8.8.2023	Negative
Recoverin antibodies (serum)	11.8.2023	Positive
	16.1.2024	Positive
	21.2.2024	Positive
	29.5.2024	Positive
Recoverin antibodies (CSF)	11.8.2023	Positive
ANCA screening	2.11.2023	Negative
Total complement activity	4.3.2024	Increased
Complement C5	30.1.2024	21.0 mg/dl (n, 9.02-6.02 mg/dl) 21.0 mg/dl (n, 9.02-6.02 mg/dl)
	29.5.2024	
Complement C9	30.1.2024	Reduced
Fibronectin	9.10.2023	59.8 mg/dl (n, 25-40 mg/dl)
	4.3.2024	52.4 mg/dl (n, 25-40 mg/dl)
Anti-GFAP	28.5.2024	Negative
S100B (serum)	4.3.2024	Normal
Zonulin (serum)	28.10.2022	Increased
NfH	16.1.2024	Normal
NfL	16.1.2024	Normal

ERG in September 2023 under scotopic and photopic conditions was normal. Quantitative sensory testing in October 2023 showed normal Aβ and Aγ fiber function but impaired Aδ fiber function in the upper extremities and was normal in the lower extremities, confirming the diagnosis of SFN. Quantitative sudomotor axon reflex showed increased latencies bilaterally. Based on clinical presentation, quantitative sensory testing, sudomotor axon reflex, skin biopsy, and CCM, SFN was diagnosed.

**Figure 2:**
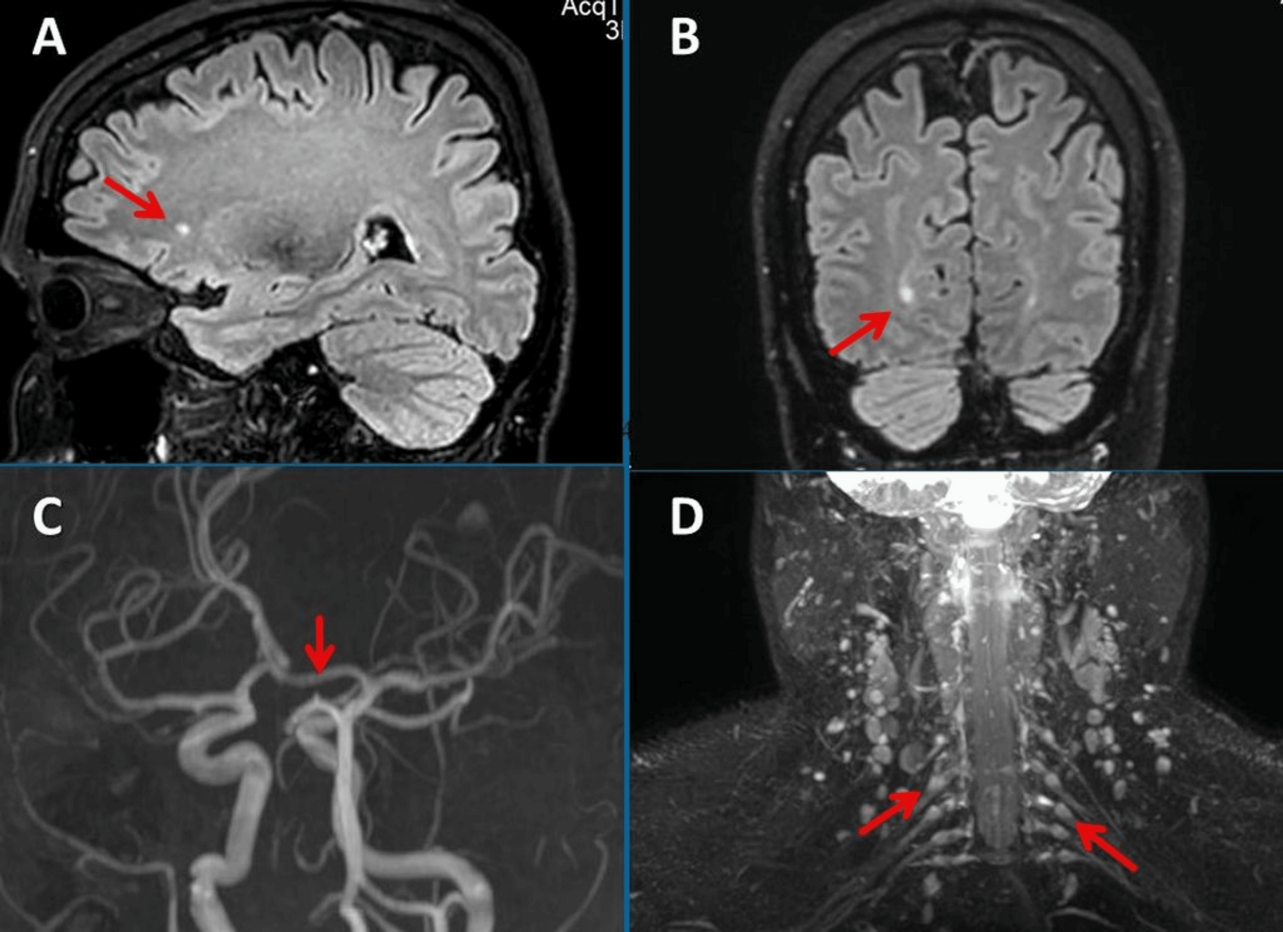
Cerebral MRI in January 2024 shows spot-like gliosis in the right frontal white matter on FLAIR (panels A and B). Time-of-flight MRA shows a small aneurysm in the P1 segment of the right posterior cerebral artery (panel C). Nerve MRI shows prominent dorsal root ganglia of the inferior cervical nerve roots, suggestive of ganglionitis (panel D) MRI, magnetic resonance imaging; FLAIR, fluid-attenuated inversion recovery; MRA, magnetic resonance angiography.

An ophthalmological examination in October 2023 revealed bilateral keratoconjunctivitis sicca, hyperopia, astigmatism, presbyopia, euchromatopsy, orthophoria, and steropsia. Funduscopy revealed the TV, KZ, and KS of the retinal arteries. Optical coherence tomography (OCT) was normal bilaterally, but OCT angiography (OCTA) revealed microcirculatory dysfunction with poor visualization of capillary blood flow on both sides. Anti-recoverin IgG antibodies were again positive in serum. MRI of the brain and cervical spine in January 2024 showed nonspecific gliotic spots, enlarged cervical lymph nodes, prominent spinal ganglions, and a stable right-sided PCA aneurysm (Figure 2).

During an outpatient neurological evaluation in February 2024, immunoadsorption or B-cell depletion was suggested to eliminate recoverin antibodies. Another neurological evaluation in May 2024 revealed elevated CSF protein and again elevated serum anti-recoverin IgG antibodies (Table 2). Anti-myelin IgG and anti-glial fibrillary acidic protein antibodies were negative (Table 2). Chronic inflammatory demyelinating polyneuropathy (CIDP) was suspected and immunoadsorption was recommended. Further results of relevant blood tests during the course of the disease are listed in Table 2.

He is currently (July 2024) on therapy with prednisolone (5-10 mg), low-dose naltrexone (5 mg), rosuvastatin (10 mg), candesartan (8 mg/day), aspirin (100 mg/d), clopidogrel (75 mg/day), gabapentin (900 mg/day), ivermectin (12 mg/day) due to increasing spike antibodies, desloratadine for suspected mast cell activation syndrome, cyclosporine eye drops, and hydrogel (7 times daily). In addition, he is currently undergoing immunoadsorption.

## Discussion

The patient is of interest because he experienced severe, persistent adverse reactions to the first dose of the BNT162b2 vaccine (PACVS), some of which have not resolved to date. The visual impairment has affected the patient the most. Initially, the symptoms were considered psychosomatic, but antidepressants were ineffective. As the complaints lasted longer, additional symptoms appeared, and the investigation became more intensive, it became increasingly clear that the complaints were indeed due to vaccine-induced multisystem inflammatory syndrome in adults [12]. Arguments for a causal relationship are that the onset of symptoms was temporally related to vaccination, that some of the adverse reactions reported by the patient have been previously reported as complications of SC2V [13], and that antibodies to recoverin, repeatedly elevated CSF protein, elevated immunoglobulins, and elevated interleukins support the abnormal immunological response to the vaccine.

Arguments for a pathophysiological role of anti-recoverin antibodies are that the patient was temporarily blind, had permanent visual impairment, amblyopia, reduced dim vision, and color vision deficiency, that anti-recoverin antibodies were repeatedly elevated in serum and cerebrospinal fluid, and that anti-recoverin antibody syndrome has been previously described as retinopathy, uveitis, and cerebellar syndrome [14]. There is also a report of a patient with optic neuropathy associated with anti-recoverin antibodies [15]. Anti-recoverin antibodies have also been described in association with retinal degeneration [16,17].

Whether the patient had mild Guillain-Barré syndrome (GBS) or CIDP remains speculative, but it is suspected that mild GBS developed five weeks after vaccination. Arguments for GBS as a manifestation of PACVS are the clinical presentation (weakness of the lower extremities leading to gait disturbances, reduced fine motor skills, myoclonus), cytoalbumin dissociation, and the fact that the association of GBS and SFN after SC2V has been repeatedly reported [11].

Although the patient had neither pleocytosis nor a contrast-enhanced lesion on MRI, it cannot be definitively ruled out that he also had encephalitis. Arguments for encephalitis include headache, photosensitivity, neck pain, and elevated protein levels in the CSF. The absence of contrast enhancement does not necessarily exclude encephalitis. A strong argument for encephalitis is that two patients with AIE in association with anti-recovery antibodies were recently reported [6,18].

Arguments for endothelialitis and endothelial dysfunction were the pain symptoms (endothelialitis causes eye pain, photophobia, perivascular edema, and visual disturbances), capillary microscopy of the nails showing microbleeds, dilatation, increased capillary branching, and abnormal laser perfusion measurement. Endothelialitis is one of the pathophysiological explanations for SC2V-associated thrombosis [19].

It remains speculative which of the abnormal findings in instrumental examinations were already present before vaccination and thus have other causes. However, since the patient was healthy before SC2V and the spondylarthritis has been inactive for 10 years, it is very likely that the clinical presentation and the abnormal instrumental findings are due to SC2V. Spondylarthritis has not been reported in association with anti-recoverin antibodies. However, there is evidence that side effects of SC2V occur more frequently in patients with an immunological history than without [20].

Limitations of the study are that the investigation of ACVS and PACVS was delayed, the causal relationship between SC2V and the patients' complaints has still not been fully proven, and that therapies were often only carried out half-heartedly and delayed.

## Conclusions

This case shows that acute and post-acute side effects can occur after SC2V with BNT162b2, which manifest themselves in cerebral (encephalopathy), ocular (retinopathy), and peripheral nerve disease (SFN), among others. These clinical manifestations may be triggered by an exaggerated immune response to SC2V, manifesting as hyperimmunoglobulinemia, elevated interleukins, TH1/TH2 shift, and BBB leakage. Although the pandemic is over, the long-term consequences of SC2V in the form of PACVS remain, which should not be neglected and should be treated accordingly in a timely manner. Future studies on the pathophysiology of PACVS are needed.
